# Clinical characteristics and survival outcomes of ascending, descending and mixed types of nasopharyngeal carcinoma in the non‐endemic areas of china: A propensity score matching analysis

**DOI:** 10.1002/cam4.3537

**Published:** 2020-10-14

**Authors:** Yixin Fan, Wenqiang Guan, Rui Huang, Stefan (YUJIE) Lin, Yanqiong Song, Shun Lu, Le Kang, Qin Yang, Jinyi Lang, Peng Zhang

**Affiliations:** ^1^ Department of Radiation Oncology Sichuan Cancer Hospital & Institute Sichuan Cancer Center School of Medicine University of Electronic Science and Technology of China Radiation Oncology Key Laboratory of Sichuan Province Chengdu China; ^2^ Graduate School Chengdu Medical College Chengdu China; ^3^ Department of Computer Science and Engineering Office for Student Affairs University of Minnesota‐Twin Cities, Economics Institute, School of Statistics Minneapolis MN USA; ^4^ Viterbi School of Engineering Applied Data Science University of Southern California Los Angeles CA USA

**Keywords:** ascending type, clinical characteristics, descending type, mixed type, nasopharyngeal carcinoma, non‐endemic area, survival outcomes

## Abstract

**Purpose:**

To compare the clinical characteristics and survival outcomes of patients with ascending type (type A), descending type (type D), and mixed type (type AD) of nasopharyngeal carcinoma (NPC) in non‐endemic areas.

**Materials and methods:**

The cohort included 628 patients diagnosed with type A, type D, and type AD of NPC between January 2009 and December 2014. Type A was defined as T_3‐4 _N_0‐1_, type D as T_0‐1 _N_2‐3_, and type AD as T_3‐4 _N_2‐3_. Propensity score matching (PSM) was performed to balance clinical factors and match patients. Kaplan‐Meier methods and Cox proportional hazards models were used to evaluate the impact of different NPC types on survival outcomes.

**Results:**

There were 145 patients with type A, 194 with type D, and 289 with type AD. However, after PSM, there were only 130 patients with each type. Compared with patients with type A, those with type D had lower 5‐year disease‐specific survival (96.9% vs 91.5%) and distant metastasis‐free survival (92.3% vs 77.7%) and higher local relapse‐free survival (88.5% vs 96.9%) (*p* < 0.05 for all). Patients with type AD may have an increased risk of disease progression (progression‐free survival, 56.9% vs 74.6% and 66.2%) and death (overall survival [OS], 76.9% vs 85.4% and 85.4%) (*p* < 0.05 for all) compared to patients with the other two types of tumors.

We further analyzed the metastasis trend. Similar metastasis patterns were observed in types AD and D, and types AD and A had similar recurrence trends. The mortality rate of patients with types AD and D in the first 3 years after metastasis was remarkably higher than that of patients with type A.

**Conclusions:**

In non‐endemic areas of China, metastases and recurrence patterns differed across tumor types. Type AD has the worst OS, and the clinical process is more radical. Type D has a lower recurrence rate, higher metastasis, and disease‐related mortality rates, and poorer prognosis after metastasis than type A.

## Graphical abstract

This article through the classification of nasopharyngeal carcinoma in non‐endemic areas, and then explore the clinical characteristics and survival results of different types.

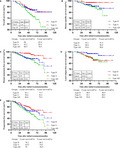

## 1 INTRODUCTION

Nasopharyngeal carcinoma (NPC) is a rare disease with obvious regional characteristics.^1^ According to the 2018 Global Cancer Statistics, approximately 129,079 new NPC cases are identified annually.^2^ Nevertheless, >70% of the new cases are in Southeast Asia, especially in Southern China,^2^ with an age‐standardized rate of 20‐40/100000 in Southern China.^3^ Sichuan Province is located in Southwest China, and numerous studies have proven that it is a non‐endemic area of NPC.^4^, ^5^ According to the provincial age‐standardized rate of NPC in China, in 2013, the incidence in Sichuan was 3.2/100000 individuals.^6^ For endemic areas, locally advanced NPC was divided into the ascending, descending, and mixed types according to the natural progression characteristics.^7^, ^8^ Sun et al^8^ used large data research methods to analyze 4252 patients with type A and 942 patients with type D and reported, in detail, the clinical characteristics and survival results of types A and D.

For the endemic areas, the aforementioned study provided detailed clinical biological behaviors and risk factors related to type A and D NPC. However, the clinical biological behaviors of the three types of NPC in non‐endemic areas remain unclear. Based on the aforementioned premise, we need to further explore whether this type of approach can better guide the prognosis of NPC cases. Therefore, we primarily analyzed the clinical characteristics and survival results of the three types of NPC in the non‐endemic areas. We used propensity score matching (PSM) and conducted a retrospective study comparing survival outcomes of patients with the three types of NPC in the non‐endemic areas. This study aimed to explore the time trend of distant metastasis and recurrence of the three types of NPC and further compare the survival after distant metastasis.

## 2 MATERIALS AND METHODS

### 2.1 Study patients

The cohort included patients with NPC who had been living in Sichuan Province for a long time and were treated at Sichuan Cancer Hospital from January 2009 to December 2014. The inclusion criteria of this study were as follows: (1) diagnosis of NPC pathologically and the 8th edition of the American Joint Committee on Cancer (AJCC) stages III–IV; (2) effective and accurate magnetic resonance imaging (MRI) and related imaging follow‐up; (3) Intensity modulated radiotherapy (IMRT) with or without chemotherapy; and (4) Karnofsky performance score ≥70. Patients with distant metastases from the first visit were excluded. The study protocol was approved by the Research Ethics Committee of the Cancer Center of Sichuan Cancer Hospital.

We used a standardized data collection form to obtain relevant information, including sex; age; family history of cancer; smoking; alcohol consumption; treatment; lactate dehydrogenase (LDH), hemoglobin (HGB), high‐sensitivity C‐reactive protein (hs‐CRP), and pretreatment lymphocyte levels; and Epstein‐Barr virus (EBV). Before diagnosis and treatment, patients underwent complete pretreatment evaluations including routine blood tests, blood biochemistry, nasopharyngoscopy, electrocardiography, MRI (scanning from the cranium to the supraclavicular fossa), radiography of the chest or contrast‐enhanced computed tomography (CT) of the chest, abdominal ultrasonography or contrast‐enhanced CT of the abdomen, whole‐body bone scintigraphy, and ^18^F‐Fluorodeoxyglucose positron emission tomography and computed tomography (PET/CT), if necessary. All patients received intensity‐modulated radiotherapy, and primary nasopharyngeal lesions (GTVnx) and metastatic cervical lymph nodes (GTVnd) were delineated according to the standards of the International Commission on Radiation Units and Measurements 50 and 62. Clinical target volumes (CTVs) were individually sketched according to tumor invasion patterns. The prescribed doses were 66‐76 Gy for GTVnx, 66‐70 Gy for GTVnd, 60 Gy for CTV‐1 (high‐risk regions), and 54 Gy for CTV‐2 (low‐risk regions). Patients had to receive one fraction daily for 5 days per week. Concurrent chemotherapy was mainly paclitaxel (120‐135 mg/m^2^, d1) and cisplatin (25 mg/m^2^, d1‐3) combination chemotherapy, repeated every 3 weeks for three cycles.

All patients had restaging according to the 8th edition of the AJCC Cancer Staging Manual.^9^ Moreover, in this study, type AD was defined as mainly advanced local disease and advanced lymph node metastasis. Type A was defined as advanced local disease and early‐stage cervical lymph‐node involvement. Type D was defined as advanced lymph node metastases but early‐stage local disease.

### 2.2 Follow‐up and outcome

After treatment, patients were evaluated every 3 months in the first 2 years and every 6 months in 2‐5 years and annually thereafter. Routine examination during follow‐up included physical examination, nasopharyngoscopy, and contrast‐enhanced MRI of the nasopharynx and neck, ultrasonography of the abdomen, and chest radiography. The median follow‐up duration was 57.4 months (range, 5.6‐112.3 months). The last follow‐up was in September 2019.

### 2.3 Endpoints and statistical analysis

The primary endpoint of our study was overall survival (OS). Distant metastasis‐free survival (DMFS), local relapse‐free survival (LRFS), progression‐free survival (PFS), and disease‐specific survival (DSS) served as the secondary endpoints. OS was defined as the time from the date of initial diagnosis to the date of death from any cause. DMFS was defined as the time from the date of the initial diagnosis to the date of the first distant metastasis. LRFS was determined as the time from the date of the initial diagnosis to the date of the first local failure. PFS was considered the time from the date of the initial diagnosis to the date of the first failure or death from any cause. DSS was defined as the time from the date of the first diagnosis of the disease to the time of disease‐related death.

Statistical analyses were conducted using SPSS, version 24. PSM was calculated by logistic regression for each patient using the following covariates: age, sex, smoking, alcohol consumption, family history of cancer, and LDH, CRP, and HGB levels. A 1:1 protocol with a caliper of 0.01 was used in matching. The Kaplan‐Meier method was used to calculate the OS, DSS, DMFS, LRFS, and PFS rates, and the log‐rank test was used to compare the survival curves among different treatment groups. In the multivariate analyses, the Cox proportional hazards regression model was used to estimate hazard ratios and 95% confidence intervals. Additionally, line charts and histograms were generated using the rms package in R version. The line chart was used to summarize the three types of recurrence and metastasis trends. The histogram shows the survival results after distant metastasis. All statistical tests were two tailed, and P‐values of less than 0.05 were considered to indicate statistical significance.

## 3 RESULTS

### 3.1 Patient characteristics in the three types of NPC

Data of patient characteristics stratified by types A, D, and AD are presented in Table 1. Among these, there were 289 patients with type AD, accounting for the largest proportion (289/628, 46%), and obviously more patients with type D (194/628, 30.9%) than patients with type A (145/628, 23.1%).

**Table 1:** Clinical characteristics and after‐PSM characteristics of patients with three types of NPC

Characteristic	Type Total data n = 145	A(No. %) After PSM n = 130	Type Total data n = 194	D(No. %) After PSM n = 130	Type Total data n = 289	AD(No. %) After PSM n = 130
Sex						
Male	94 (64.8%)	88 (67.7%)	134 (69.1%)	85 (65.4%)	200 (69.2%)	94 (72.3%)
Female	51 (35.2%)	42 (32.3%)	60 (30.9%)	45 (34.6%)	89 (30.8%)	36 (27.7%)
Age, y						
<50	77 (53.1%)	74 (56.9%)	123 (63.4%)	77 (59.2%)	178 (61.6%)	69 (53.1%)
≥50	68 (46.9%)	56 (43.1%)	71 (36.6%)	53 (40.8%)	111 (38.4%)	61 (46.9%)
Overall stage (8th edition)						
III	57 (39.3%)	53 (40.8%)	136 (70.1%)	90 (69.2%)	91 (31.5%)	40 (30.8%)
IV	88 (60.7%)	77 (59.2%)	58 (29.9%)	40 (30.8%)	198 (68.5%)	90 (69.2%)
Smoking						
Yes	56 (38.6%)	45 (34.6%)	66 (34%)	47 (36.2%)	102 (35.3%)	53 (40.8%)
No	89 (61.4%)	85 (65.4%)	128 (66%)	83 (63.8%)	187 (64.7%)	77 (59.2%)
Alcohol consumption						
Yes	39 (26.9%)	29 (22.3%)	41 (21.1%)	30 (23.1%)	62 (21.5%)	34 (26.2%)
No	106 (73.1%)	101 (77.7%)	153 (78.9%)	100 (76.9%)	227 (78.5%)	96 (73.8%)
Family history of cancer						
Yes	17 (11.7%)	15 (11.5%)	20 (10.3%)	12 (9.2%)	25 (8.7%)	11 (8.5%)
No	128 (88.3%)	115 (88.5%)	174 (89.7%)	118 (90.8%)	264 (91.3%)	119 (91.5%)
T staging						
T1			32 (16.5%)	25 (19.2%)		
T2			162 (83.5%)	105 (80.8%)		
T3	57 (39.3%)	53 (40.8%)			131 (45.3%)	61 (46.9%)
T4	88 (60.7%)	77 (59.2%)			158 (54.7%)	69 (53.1%)
N staging						
N0	16 (11%)	12 (9.2%)				
N1	129 (89%)	118 (90.8%)				
N2			136 (70.1%)	90 (69.2%)	206 (71.3%)	83 (63.8%)
N3			58 (29.9%)	40 (30.8%)	83 (28.7%)	47 (36.2%)
HGB level, g/L						
<150	105 (72.4%)	98 (75.4%)	143 (73.7%)	93 (71.5%)	219 (75.8%)	97 (74.6%)
≥150	40 (27.6%)	32 (24.6%)	51 (26.3%)	37 (28.5%)	70 (24.2%)	33 (25.4%)
Hs‐CRP level, g/mL						
<10	130 (89.7%)	116 (89.2%)	175 (90.2%)	120 (92.3%)	254 (87.9%)	118 (90.8%)
≥10	15 (10.3%)	14 (10.8%)	19 (9.8%)	10 (7.7%)	35 (12.1%)	12 (9.2%)
LDH level, U/L						
<250	141 (97.2%)	126 (96.9%)	185 (95.4%)	125 (96.2%)	269 (93.1%)	123 (94.6%)
≥250	4 (2.8%)	4 (3.1%)	9 (4.6%)	5 (3.8%)	20 (6.9%)	7 (5.4%)
Treatment modality						
RT alone	2 (1.4%)	2 (1.5%)	1 (0.5%)	1 (0.8%)	6 (2.1%)	3 (2.3%)
CCRT alone	73 (50.3%)	65 (50%)	90 (46.4%)	59 (45.4%)	138 (47.7%)	64 (49.2)
NACT plus RT	2 (1.4%)	2 (1.5%)	1 (0.5%)	1 (0.8%)	6 (2.1%)	3 (2.3%)
NACT plus CCRT	68 (46.9%)	61 (47%)	102 (52.6%)	69 (53%)	139 (48.1%)	60 (46.2%)

Abbreviations: CCRT, concurrent chemoradiotherapy; HGB, hemoglobin; Hs‐CRP, high‐sensitivity C‐reactive protein; LDH, lactate dehydrogenase; NACT, neoadjuvant chemotherapy; NPC, nasopharyngeal carcinoma; PSM, propensity score matching; RT, radiotherapy.

### 3.2 Survival and multivariate analyses of three types of NPC

**Figure 1:**
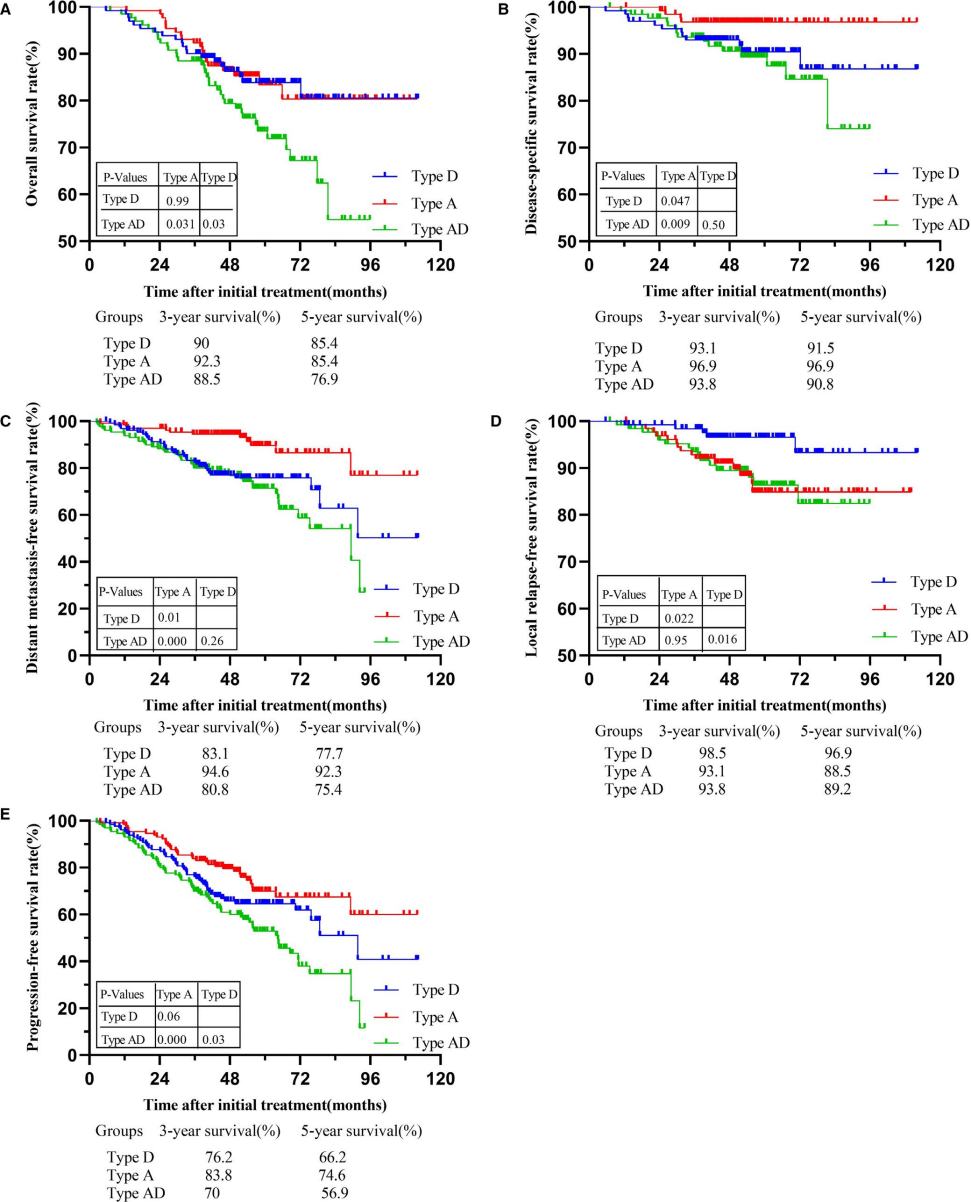
Kaplan–Meier's survival curves are shown for (A) overall survival, (B) disease‐specific survival, (C)distant metastasis‐free survival, (D) local relapse‐free survival,and (E) disease‐free survival in patients with type A, type D and type AD NPC.

First, three types of NPC were matched by PSM. There were 130 cases in each group after matching. Patient features stratified after PSM by types A, D, and AD are presented in Table 1. Patients with type A and D tumors had similar 5‐year OS rates (85.4% vs 85.4%; *p* > 0.05; Figure 1A), but patients with type D tumors were more likely to have a disease‐related death within 5 years than patients with type A tumors (DSS, 91.5% vs 96.9%; *p* < 0.05; Figure 1B). Patients with type D tumors had a higher risk of metastasis within 5 years (DMFS, 77.7% vs 92.3%; *p* < 0.05; Figure 1C) than patients with type A tumors, while there was a smaller proportion of patients with type D tumors who had local recurrence (LRFS, 88.5% vs 96.9%; *p* < 0.05; Figure 1D). Type D tumors were associated with a worse 5‐year PFS than type A tumors (66.2% vs 74.6%; *p* > 0.05; Figure 1E). Thus, compared with patients with type D tumors, those with type A tumors have significantly better DSS and DMFS. Compared with patients with the two other types, those with type AD tumors had worse 5‐year OS and PFS (*p* < 0.05; Figure 1A,E). Patients with type AD and D tumors were more likely to have disease‐related death within 5 years than those with type A tumors (*p* < 0.05; Figure 1B). Interestingly, patients with type D and AD tumors had a similar 5‐year DMFS (77.7% vs 75.4%; *p* > 0.05; Figure 1C); type AD and A tumors were similarly associated with worse 5‐year LRFS (89.2% vs 88.5%; *p* > 0.05; Figure 1D). Overall, patients with type AD had the worst prognosis. Multivariate analysis of type A tumors showed that T stage was an independent prognostic factor for OS and PFS (*p* < 0.05; Table 2). Respective multivariate analyses suggest that the N stage of type D was an independent risk factor for OS and PFS (*p* < 0.05). Moreover, T and N stages and hs‐CRP level had a strong correlation with DMFS (*p* < 0.05; Table 2). T and N stages and a family history of cancer in type AD tumors were significantly strongly associated with OS (*p* < 0.05). HGB level and sex were independent prognostic factors for LRFS (*p* < 0.05). The independent risk factors for DMFS were N stage, HGB level, and smoking (*p* < 0.05). N stage and family history of cancer had a strong correlation with PFS (*p* < 0.05; Table 2).

**Table 2:** Multivariate analysis of prognostic factors in patients with three types of NPC

Endpoint	Variable	Type A (n = 145)			Type D (n = 194)			Type AD (n = 289)		
		*p*	HR	95% CI	*p*	HR	95% CI	*p*	HR	95% CI
OS	T stage	.05	2.89	0.97,8.62				.00	1.98	1.20,3.25
	N stage				.02	2.53	1.16,5.55	.02	1.78	1.09,2.88
	Cancer history							.03	2.18	1.10,4.33
LRFS	T stage									
	N stage									
	Sex							.04	2.29	1.01,5.13
	HGB							.04	2.25	1.05,4.80
DMFS	T stage				.03	0.47	0.24,0.92			
	N stage				.00	2.40	1.38,4.41	.00	2.65	1.64,4.27
	HGB							.03	0.50	0.27,0.95
	Hs‐CRP				.04	2.40	1.02,5.68			
	Smoking							.02	1.94	1.14,3.32
PFS	T stage	.01	2.80	1.28,6.14						
	N stage				.00	2.68	1.62,4.44	.00	2.02	1.41,2.91
	Cancer history							.03	1.79	1.06,3.03

Abbreviations: CI, confidence interval; HGB, hemoglobin; HR, hazard ratio; Hs‐CRP, high‐sensitivity C‐reactive protein; NPC, nasopharyngeal carcinoma.

### 3.3 Time of metastasis and recurrence in the three types of NPC

**Figure 2:**
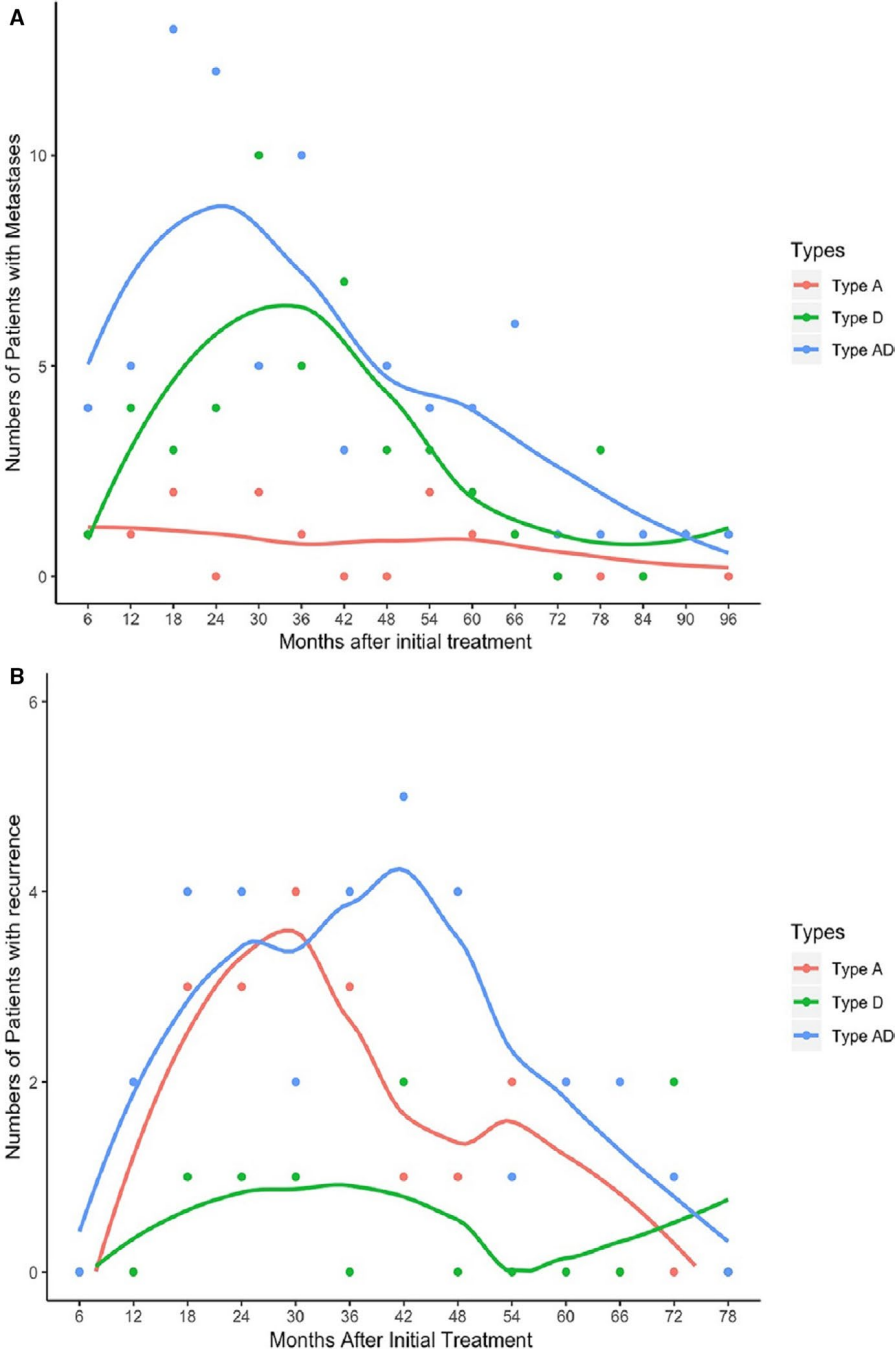
Time curves of disease metastasis (A), recurrence (B) in type A, type D and type AD NPC.

The metastasis and recurrence curves of the groups by types are shown in Figure 2A,B, respectively. When we evaluated the tumor metastasis and recurrence curves, we used a 6‐month interval. There were significantly more patients with metastasis than with recurrence. Regarding the metastasis curve, similar patterns were observed in type AD and D tumors. The number of patients with metastasis showed an upward trend and peaked at 18‐24 months and 30‐36 months after initial treatment, respectively, followed by a prolonged downtrend. In the recurrence curve, type AD and type A tumors had similar trends. The number of patients with recurrence increased and peaked at 36‐42 months and 24‐30 months after initial treatment, followed by a continuous decline.

### 3.4 Survival of patients after metastases in three types of NPC

**Figure 3:**
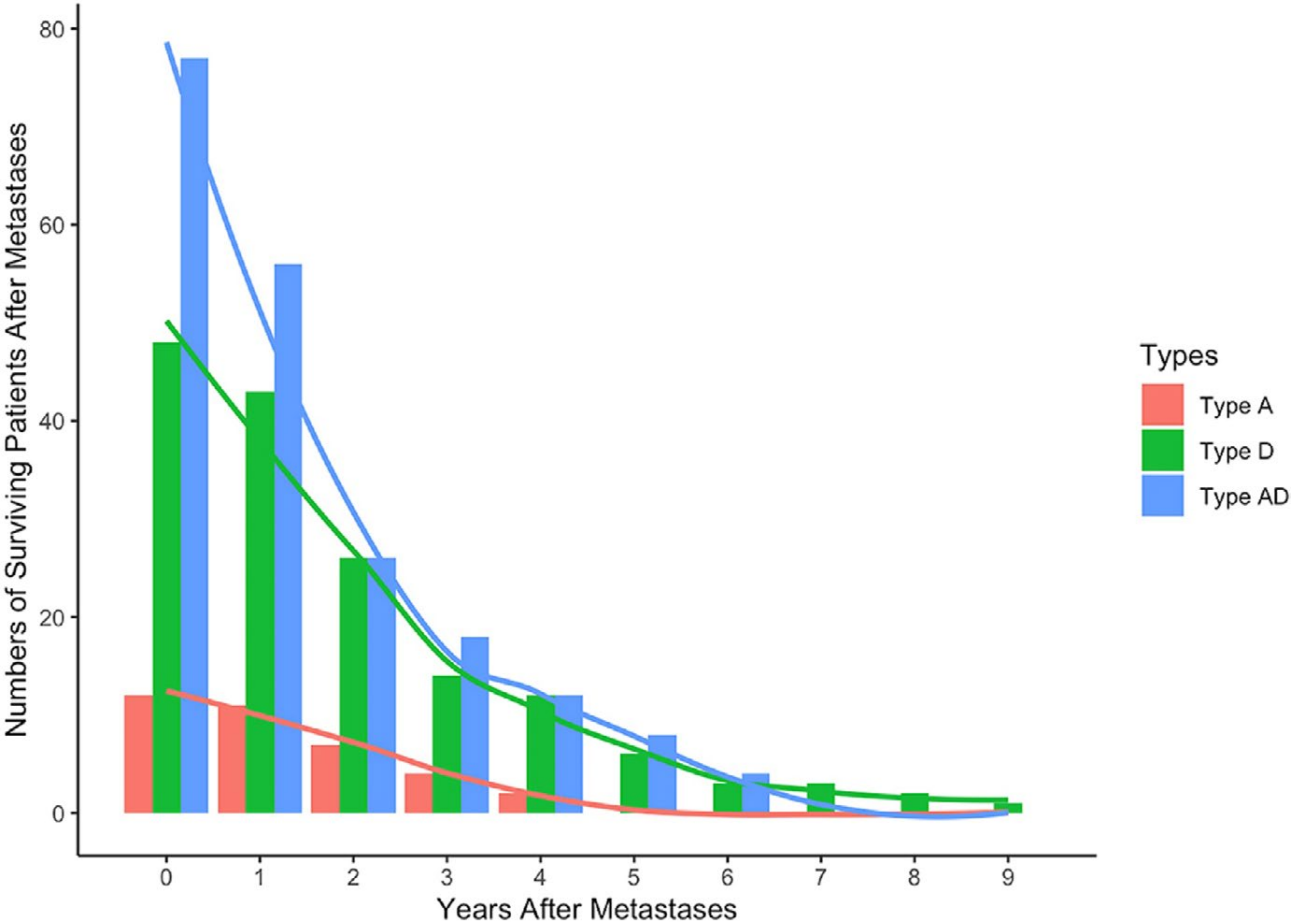
Survival curves after metastases for type A, type D and type AD NPC.

Survival for the three types of NPC after metastasis is shown in Figure 3. It is remarkable that the high mortality period of patients after metastasis is mainly concentrated in the first 3 years after metastasis. The mortality rates in patients with type D tumors (70.8%) in the first 3 years after metastasis were remarkably higher than those in patients with type A tumors (66.7%). In patients with type AD tumors, the mortality rates (76.6%) in the first 3 years after metastasis were significantly higher than those in patients with type D tumors. After 3 years, the mortality rates of patients with types D and AD were significantly reduced, and the survival trend was quite similar.

## 4 DISCUSSION

To the best of our knowledge, this is the largest clinical retrospective study in non‐endemic areas that compared the three types of NPC in 628 enrolled with stage III–IV patients stratified by clinical characteristics, survival outcomes, recurrence patterns, and metastasis. Consistent with previous studies conducted in endemic areas,^10^ type AD accounts for the largest proportion of locally advanced NPC. Different from the results in the endemic areas,^8^ the number of patients with type D tumors is larger than that of patients with type A tumors. We used the PSM method in our study to exclude possible clinical interference factors. Our study results were consistent with the findings of studies conducted in numerous endemic areas.^7^, ^8^ Type A tumors were more likely to recur, and type D tumors were more likely to metastasize. In endemic areas, patients with type D tumors have a worse 5‐year OS than patients with type A tumors.^8^ In non‐endemic areas, patients with type D tumors have a worse 5‐year DSS than those with type A tumors. The highlight of our study was that type AD and A tumors had similar 5‐year LRFS and recurrence trend, and the 5‐year DMFS and metastasis patterns of type D tumors were similar to those of type AD tumors. Furthermore, we focused on analyzing the relationship between type A tumors and recurrence rates of stage III and IV. The recurrence rate of type A tumors was significantly higher than that of stage III tumors (*p* < 0.05; Supplementary Figure S1), and there was no significant difference from that of stage IV tumors (*p* > 0.05). Furthermore, we also focused on the relationship between metastasis rates in type D tumors and stage III–IV. The proportion of 5‐year DMFS in stage Ⅲ tumors was higher than that in type D tumors (*p* < 0.05; Supplementary Figure S2), and there was no significant difference between the 5‐year DMFS in type D tumors and stage IV tumors (*p* > 0.05). Therefore, we should pay more attention to the recurrence rate in type A tumors and metastasis rate in type D tumors and take earlier effectively active measures.

The degree of cervical lymph node metastasis is positively related to the distant metastasis of NPC.^11^ Distant metastasis is mainly due to the fact that metastatic lymph nodes can fuse into huge masses, often compressing the internal jugular vein, forming tumor thrombus in the bloodstream.^12^ Multivariate analysis suggests that N stage was an independent prognostic factor for OS, DMFS, and PFS in type D tumors. Yao et al.^13^ pointed out that, compared with neoadjuvant chemotherapy (NACT) + concurrent chemoradiotherapy (CCRT), CCRT is significantly associated with deterioration of 5‐year DMFS. However, our study does not show any difference between the two treatment methods. EBV is a particularly sensitive indicator of lymph node metastasis in NPC. In the endemic areas of China, positive EBV accounts for the vast majority.^8^, ^14^, ^15^ Of interest though, in our available data, most patients with NPC had a negative EBV result. It has been reported in numerous studies that the incidence of EBV‐associated NPC appears to be related geographically ^1^, ^15^, ^16^. The study conducted by Xu et al^1^ found that the BALF2‐CCT gene is a highly pathogenic EBV subtype associated with NPC and shows the strongest association with the region. The infection rate of the BALF2‐CCT gene in endemic areas is >80%, and the infection rate in the non‐endemic areas of China is <5%. This may be the reason why most EBV results in the non‐endemic areas are negative, but the EBV subtype and optimal treatment of NPC need to be further studied in the non‐endemic areas. However, we believe that increasing the number of chemotherapy cycles may have a positive effect on distant metastasis. In addition, elevated hs‐CRP levels demonstrated negative prognostic indicators in patients with type D NPC. Studies^17^, ^18^ have confirmed that inflammation promoted the initiation, progression, and metastasis of cancer. Xia et al^19^ reported that baseline CRP level may be useful in predicting the prognosis of patients with metastatic NPC. However, recent studies showed that the CRP level during treatment had no relationship with DMFS in patients with NPC.^20^ A prospective study is still needed to validate this.

The typical features of type A tumors are that the primary lesion has a large range of invasion. We analyze the following: on the one hand, we believe that the development of this type may be due to long‐term invasion of the primary lesion but stage N is relatively stable; on the other hand, the T stage of this type is more aggressive and progresses rapidly. However, we do not have a clear proof, and further experiments are needed to clarify its molecular mechanism. Local recurrence is the main cause of treatment failure in patients with type A NPC, in approximately 10%.^21^ In a study on the endemic areas of China,^22^, ^23^ the proportion of recurrence in the first 2 years of the observation was 41%, and the recurrence rate in 3‐5 years was 44%. In our study, patients with type A tumors reached the first peak of recurrence in the 24th‐30th month after the initial treatment. Therefore, improving the local control of type A tumors is a critical method to increase the survival rate. NACT plus radiotherapy (RT) can significantly improve the prognosis of type A NPC.^13^ Several studies^22^, ^23^ have found that NPC recurrence was noted in the high‐dose area of RT. Kong et al^24^ suggested that most recurrences were caused by radioresistance. High‐dose irradiation with IMRT can achieve disease recurrence control, but fatal complications make treatment ineffective.^25^ Particle therapy such as proton or carbon ion RT may be a better choice because it provides unique physical properties and has a limited range of dose delivery.^24^ Induction chemotherapy is usually performed during re‐irradiation because it may reduce the volume of recurrent tumors and it is easier to retain adjacent vital organs and eliminate micrometastases.^21^ Immunotherapy including pembrolizumab and nivolumab has achieved excellent results in the second‐line treatment of NPC recurrence.^26^, ^27^ Re‐irradiation in combination with immunological checkpoint inhibitors for the treatment of recurrent NPC is currently being studied.^25^

Type AD tumors have the highest incidence in the non‐endemic areas. As the socioeconomic status of Sichuan is relatively behind and is a non‐endemic area of NPC, individuals are less aware of NPC. In addition, due to the uniqueness of the type AD gene, it can combine the characteristics of type A and D with strong invasiveness. Type AD tumor has a more aggressive clinical course and worse outcomes compared with type A and D tumors, and the peak of metastasis and recurrence occurs within 5 years after the initial treatment. The mortality rate in type AD tumors in the first 3 years after metastasis is significantly higher than that in type D and A tumors. Currently, the preferred treatment for metastatic and recurrent NPC is a platinum‐based double‐line chemotherapy regimen based on the combination of cisplatin and gemcitabine.^28^ Zhao et al^29^ demonstrated that nimotuzumab‐cisplatin and 5‐fluorouracil combination chemotherapy has potential efficacy and is well tolerated as a first‐line chemotherapy regimen in metastatic and recurrent NPC. Recently, the application of immunotherapy for the treatment of metastatic and recurrent NPC has been gradually developed and popularized, and its efficacy has been proven in the clinic. It is a promising strategy.^30^

There are some notable limitations in our study. This study primarily aimed to explore the clinical features and survival outcomes of the three types of NPC, such that there is no deep exploration of the most suitable treatment options for the three types. Moreover, a major concern in the present study is the retrospective nature of the analysis from a single center among the non‐endemic population, and no external validation is performed.

## DATA AVAILABILITY STATEMENT

The data that support the findings of this study are available from the corresponding author upon reasonable request.

## CONFLICTS OF INTEREST

The authors declare no conflict of interest.

## AUTHOR CONTRIBUTIONS

Peng Zhang and Yixin Fan designed this study. Yixin Fan, Wenqiang Guan, Rui Huang, Stefan (YUJIE) Lin,Yanqiong Song, Shun Lu, Le Kang, Qin Yang conducted the study and analyzed the results and drafted the manuscript under the supervision of Jinyi Lang and Peng Zhang. Peng Zhang and Yixin Fan revised the manuscript finally.

## Supporting information

Fig S1

Fig S2
