# Supplementary figures and images for: Clinical characteristics and survival outcomes of ascending, descending and mixed types of nasopharyngeal carcinoma in the non‐endemic areas of china: A propensity score matching analysis
Source: Cancer Med. 2020 Oct 14;9(24):9315–25. doi: 10.1002/cam4.3537 (PMC7774743; doi:10.1002/cam4.3537)

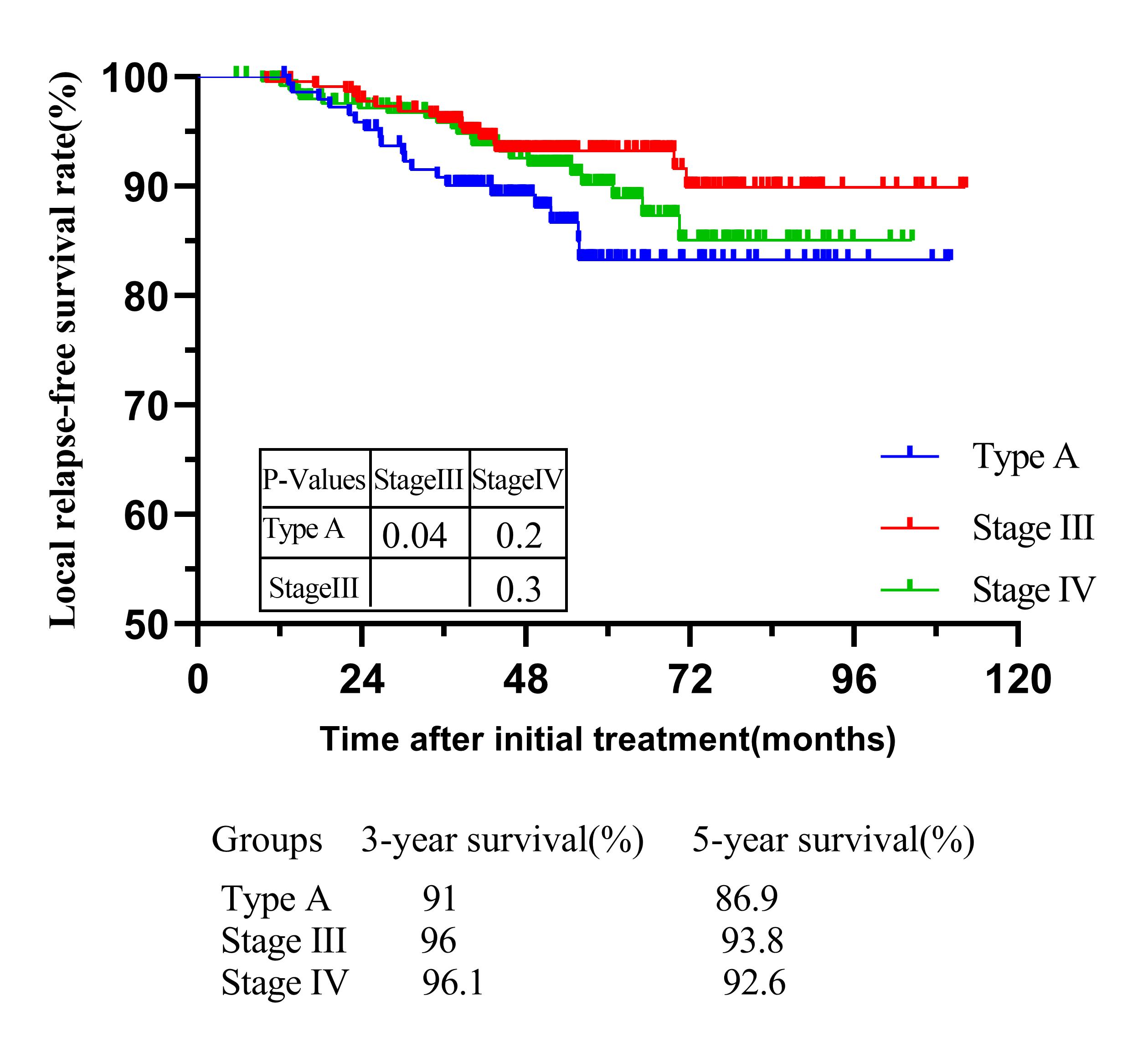

Supplement: Supplementary file 1 — Fig S1 [file CAM4-9-9315-s001.jpg]

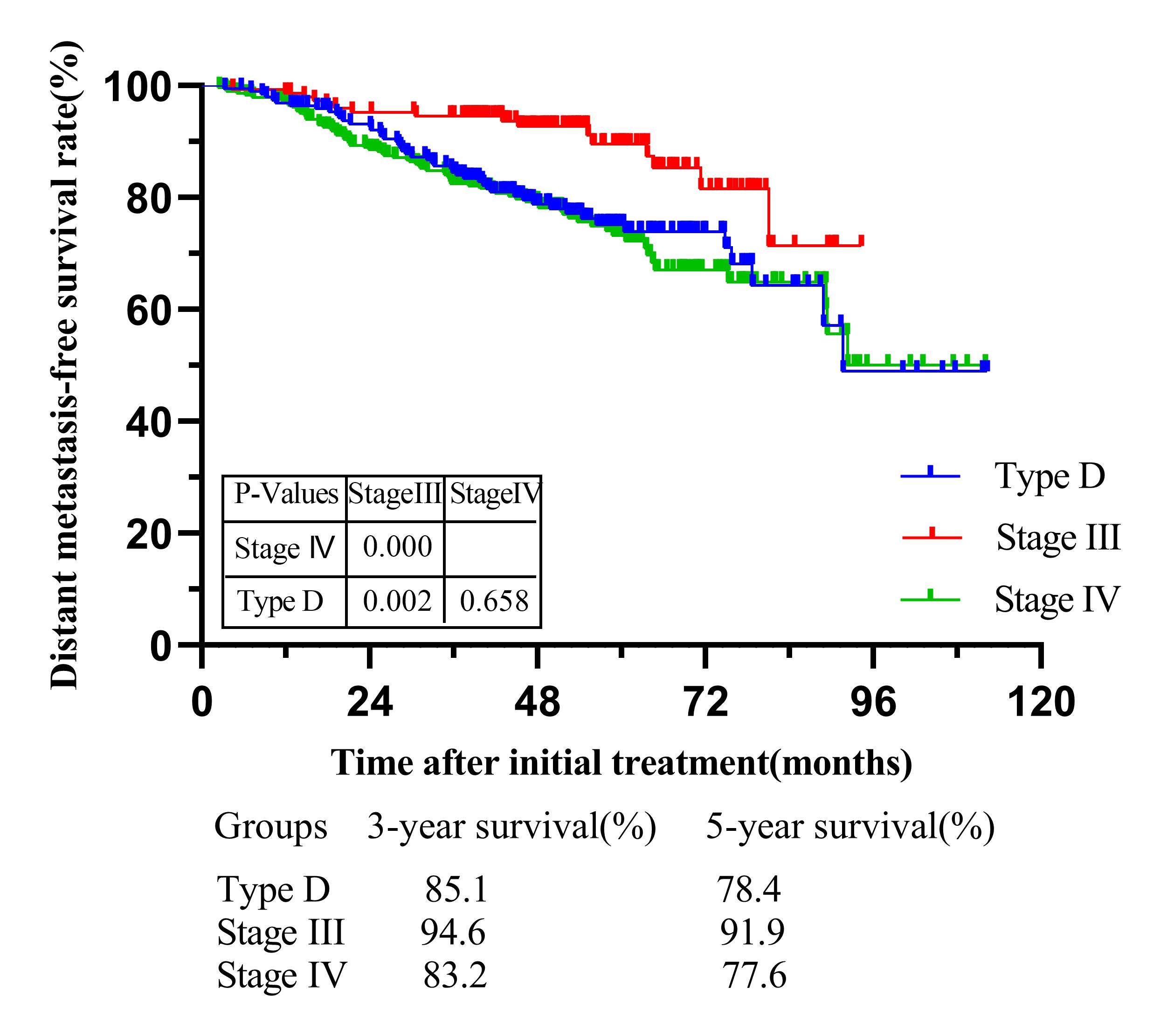

Supplement: Supplementary file 2 — Fig S2 [file CAM4-9-9315-s002.jpg]
